# Natural history and treatment of hepatitis B virus and hepatitis C virus coinfection

**DOI:** 10.1186/1476-0711-4-13

**Published:** 2005-09-13

**Authors:** Seth D Crockett, Emmet B Keeffe

**Affiliations:** 1 Department of Medicine, Stanford University School of Medicine, Stanford, California, USA; 2 Division of Gastroenterology and Hepatology, Stanford University School of Medicine, Stanford, California, USA

**Keywords:** Treatment, Hepatitis B, Hepatitis C, HBV/HCV, Coinfection, Dual infection, Interferon, Ribavirin, Lamivudine, Triple infection, HBV/HCV/HDV, HBV/HCV/HIV

## Abstract

Hepatitis B virus (HBV) and hepatitis C virus (HCV) coinfection is not uncommon as a result of similar routes of infection. Patients who are coinfected represent a unique group with diverse serologic profiles. Combined chronic hepatitis B and C leads to more severe liver disease and an increased risk of hepatocellular carcinoma. Furthermore, coinfected patients represent a treatment challenge. No standard recommendations exist for treatment of viral hepatitis due to dual HBV/HCV infection, and therefore treatment must be individualized based on patient variables such as serologic and virologic profiles, patient's prior exposure to antiviral treatment, and the presence of other parenterally transmitted viruses such as hepatitis D virus and human immunodeficiency virus. The natural history and treatment of patients with HBV and HCV coinfection is reviewed.

## Introduction

Hepatitis B and hepatitis C viruses are the most common causes of chronic liver disease worldwide. Acute infection with hepatitis B virus (HBV) or hepatitis C virus (HCV) may result in chronic infection, which occurs at a high rate in infants infected with HBV and the majority of individuals infected with HCV. Chronic HBV and/or HCV infection can progress to cirrhosis and be complicated by hepatocellular carcinoma (HCC). Coinfection with both viruses can occur because of shared routes of infection. Patients with dual HBV and HCV infection have more severe liver disease, and are at an increased risk for progression to HCC [1-4]. Coinfected patients represent a diverse group with various viral replication and immunity profiles. Because of their distinct clinical course and heterogeneity, identification of patients who are candidates for therapy and selection of the optimal antiviral therapy is a challenge for clinicians. Herein we review the natural history of HBV/HCV coinfection, current understanding of the interactions between these hepatotropic viruses, and the limited literature on treatment of coinfected patients.

### Epidemiology

Approximately 350 million people are infected with HBV worldwide [5], and the World Health Organization estimates that approximately 170 million people are infected with HCV [6]. The exact number of patients infected with both HCV and HBV is unknown. One Eastern European study found a rate of dual infection in 0.68% of a randomly selected healthy population of over 2200 individuals [7]. In patients with chronic hepatitis B, estimates of the rates of HCV coinfection vary from 9% to 30%, depending on the geographic region [8]. One Italian study found that rates of dual infection increased with age, and was more common in patients over 50 years of age [9]. These numbers may underestimate the true number of patients with both viral infections because no large-scale studies have been performed, and there is a well-described phenomenon of "serologically silent" occult HBV infection (i.e. patients with negative hepatitis B surface antigen [HBsAg] but detectable serum HBV DNA) in patients with chronic hepatitis C [10].

### Screening for Coinfection

Persons with a first episode of acute hepatitis should be screened for all viral causes including HBV and HCV. Some patients may be inoculated with both viruses simultaneously and will present with acute hepatitis due to both viruses. In addition, HBV superinfection in patients with chronic hepatitis C, and HCV superinfection in patients with chronic hepatitis B have both been reported [11-13]. Therefore, episodes of acute hepatitis in patients with known chronic HBV or HCV infection, especially those with ongoing risk behavior for infection with the alternative virus such as injection drug users, should raise suspicion and prompt screening for superinfection. In addition, as will be described below, silent or occult HBV infection in patients with chronic hepatitis C may alter patients' clinical course and response to therapy [14,15]. However, this phenomenon requires further study regarding its clinical significance before routine screening of HCV patients for HBV DNA can be recommended.

### Interaction of Hepatitis Viruses

Several studies have shown that the HBV and HCV interact with each other and affect immune responses. HCV infection can suppress HBV replication, as demonstrated by studies showing that patients with chronic hepatitis B who are coinfected with HCV have lower HBV DNA levels, decreased activity of HBV DNA polymerase, and decreased expression of HBsAg and hepatitis B core antigen in the liver [16-18]. Furthermore, patients with chronic HBV infection who become superinfected with HCV can undergo seroconversion of hepatitis B e antigen (HBeAg) and HBsAg to respective antibodies [19-21]. Sheen et al. [22] conducted a longitudinal follow-up study of a large series of HBV infected patients and found that the annual incidence of HBsAg seroconversion was 2.08% in coinfected patients compared to 0.43% in patients with HBV monoinfection, and a subsequent study confirmed these results [23]. Several mechanisms of replicative interference of HBV by HCV have been proposed. Shih et al. implicated the hepatitis C core protein in suppression of HBV [24]. A subsequent study found that the hepatitis C core protein suppressed HBV enhancer activity, thereby affecting transcription [25]. This inhibitory effect appears to be more pronounced with HCV genotype 1 both *in vitro *and *in vivo *[25,26].

Several authors have reported that HBV can reciprocally inhibit HCV replication as well [27,28]. Specifically, HBV DNA replication has been shown to correlate with decreased HCV RNA levels in coinfected patients [29]. In one Italian study, coinfected patients had a rate of HCV RNA clearance of 71% compared to 14% with HCV monoinfection [26]. HBV replication in coinfected individuals may result in more liver inflammation, as demonstrated by studies in which HBV replication correlated with elevated ALT levels, while HCV replication did not [28,30,31]. Furthermore, coinfected patients have been demonstrated to have lower levels of both HBV DNA and HCV RNA than corresponding monoinfected controls, indicating that concurrent suppression of both viruses by the other virus can also occur. [32].

Overall, the available evidence demonstrates that both viruses can inhibit each other simultaneously; either virus can play a dominant role; both viruses have the ability to induce seroconversion of the other; the chronology of infection has a role in determining the dominant virus; and HBV and HCV can alternate their dominance [8]. However, the overall dominant effect appears to be HCV suppression of HBV [11,32,33].

### Clinical Scenarios

**Figure 1:**
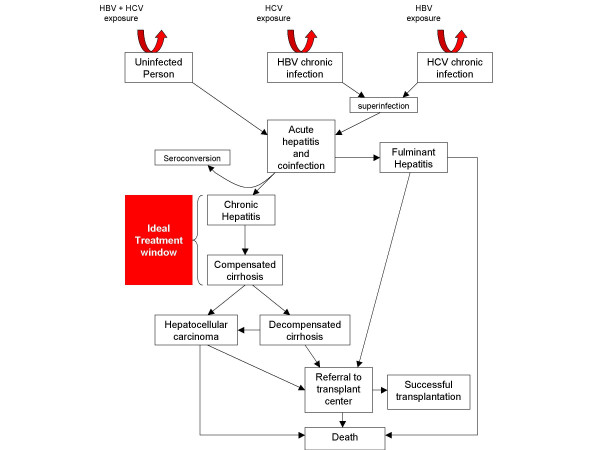
Infectious scenarios and treatment window for patients coinfected with hepatitis B and hepatitis C viruses. HBV = hepatitis B virus, HCV = hepatitis C virus.

Different scenarios of infection have been described with dual infection with HBV and HCV including acute dual viral hepatitis, occult HBV coinfection of chronic hepatitis C, and superinfection by either virus in patients with preexisting chronic hepatitis due to the alternative virus. In addition, coinfected patients are often found to have evidence of both HBV and HCV infection without a clear chronology of infection. In areas with high endemic rates of HBV infection due to vertical transmission, coinfection can generally be assumed to be due to HCV superinfection. In other geographic areas, the sequence of infections is less clear. Acute coinfection or superinfection with either virus can lead to fulminant hepatitis, chronic hepatitis, cirrhosis and HCC (Figure 1).

#### Acute Hepatitis due to Dual Infection

Few studies have been reported on simultaneous acute hepatitis with HBV and HCV, but it appears that HBV and HCV interact in acute infections similar to their interactions in chronic infection. Simultaneous coinfection via an accidental needle stick that resulted in acute hepatitis was first described by Liaw in 1982 [34]. The diagnosis of HBV infection was delayed for 6 weeks, possibly related to HCV interference. Simultaneous coinfection resulting in acute post-transfusion hepatitis has also been described [35]. Mimms et al [36]. studied patients with concurrent acute infection with HBV and HCV compared to those with acute HBV alone, and reported decreased ALT levels, delayed appearance of HBsAg, and a shorter duration of hepatitis B surface antigenemia in coinfected patients compared to controls, suggesting HCV suppression of HBV activity.

#### Occult Hepatitis B Infection

Several reports have described the entity of occult or serologically silent HBV infection, particularly as it relates to treatment outcomes [10,14]. Occult HBV infection refers to patients who have low levels of circulating HBV DNA, but who lack both circulating antigens and their corresponding antibodies (i.e. HBsAg, HBeAg, hepatitis B surface antibody [anti-HBs] and hepatitis B e antibody [anti-HBe]). Such patients have been shown to have more severe liver disease [37]. One study found that 33% of patients with chronic hepatitis C and occult HBV infection had cirrhosis, compared to 19% of patients with chronic HCV infection without detectable HBV DNA [38]. Occult HBV may also be associated with more liver inflammation, greater histological activity of hepatitis, and higher ALT levels [14]. The effect of serologically silent infections on response to treatment will be discussed below.

#### Hepatitis C Superinfection

In areas of high prevalence of HBV infection such as Asian countries, the phenomenon of HCV superinfection is well described [11,13]. Superinfection with HCV can result in suppression of HBV replication, as well as termination of the HBsAg carriage [11]. There is one case report of acute HCV superinfection resulting in HBeAg seroconversion as well as clearance of HBsAg [20]. After superinfection and seroconversion of HBsAg, HCV infection may persist and result in continued chronic hepatitis [19]. Besides its viral interaction with HBV, HCV superinfection can result in more severe liver disease and an increased risk of fulminant hepatitis [39]. The mortality rate of HCV superinfection in chronic hepatitis B patients may be as high as 10% [11].

#### Hepatitis B Superinfection

Superinfection with HBV in patients with chronic hepatitis C is less common. Liaw et al. reported a series of 2 patients with chronic hepatitis C with documented HBV superinfection (positive IgM anti-HBc) [12]. One 82-year-old patient who was positive for both HBV DNA and HCV RNA on admission died of fulminant hepatic failure. The second patient was HBsAg positive, but HBV DNA negative. She subsequently recovered from acute hepatitis with normalization of ALT and seroconversion of HBsAg, as well as disappearance of anti-HCV antibody, indicating a possible suppressive role of HBV on HCV infection. It is difficult to draw conclusions from this limited report, but it is reasonable to assume that there may be an increased risk of fulminant hepatitis with HBV superinfection, and that those who recover are at risk for chronic coinfection. The clearance of anti-HCV in this case suggests that superinfection may allow HBV to play the dominant role in suppression of HCV.

#### Fulminant Hepatitis

Several studies have addressed the role of coinfection with HBV and HCV in fulminant hepatitis. Chu et al. [39] conducted a prospective study of patients admitted with acute hepatitis C in Taiwan. Eleven patients had fulminant hepatitis, and of these, 23% had underlying chronic HBV infection, compared to 2.9% for patients without fulminant hepatitis. This corresponded to an odds ratio (OR) for chronic HBV patients developing fulminant hepatitis with HCV superinfection of 10.2 (95% confidence interval 4.7–21.9, p < 0.01). A French study of 40 patients with fulminant and subfulminant hepatitis found that 5 of 40 patients (12.5%) had acute coinfection with HBV and HCV and 3 of 40 (7.5%) had superinfection of HCV [40]. Another Taiwanese study of 25 subfulminant and fulminant hepatitis cases found similar rates of coinfection (9.4%) and HCV superinfection of chronic hepatitis B (3.1%) [41]. These studies suggest an increased risk of fulminant hepatitis with HCV and HBV coinfection and superinfection.

#### Chronic Hepatitis

There are various immune profiles of dually infected patients with chronic hepatitis, and their immune profiles have a bearing on the choice of treatment. One possibility is dually active HBV and HCV, in which patients have detectable serum HBV DNA and HCV RNA. It stands to reason that these patients are at highest risk of progression to cirrhosis and decompensated liver disease, and therefore, should be considered for treatment. Another possibility is active HCV infection (positive HCV RNA) in the setting of an inactive HBsAg carrier. Such patients behave similar to patients with HCV monoinfection, and likely exhibit HCV viral suppression of HBV activity. Another possibility is active HBV infection in patients with inactive or prior HCV infection (HBV DNA positive/HBeAg positive/HCV RNA negative/anti-HCV positive). This immune profile is less common, and indicates HBV suppression of HCV. The various immune profiles have significance in regards to treatment options as will be discussed below (Table 3).

**Table 3:** Immune Profiles of Patients with Chronic Hepatitis due to Coinfection, and Suggested Treatments Based on Published Trials

**Immune profile**	**HBV DNA**	**HBsAg**	**HBeAg**	**HCV RNA**	**Anti-HCV**	**Possible treatments [ref]**
Dually active	+	+/-	+/-	+	+	IFN alone [54] IFN + ribavirin [57-59] IFN + lamivudine [61]
Active HCV in HBV carrier	-	+	-	+	+	IFN alone [23, 53, 55] IFN + ribavirin [57-59]
Active HBV in chronic HCV	+	-	-	+	+	IFN alone [10] IFN + lamivudine [61]
Silent HBV	+	-	-	+/-	+	IFN alone [10] IFN + lamivudine [61]

Abbreviations: HCV = hepatitis C virus; HBV = hepatitis B virus; DNA = deoxyribonucleic acid; RNA = ribonucleic acid; HBsAg = Hepatitis B surface antigen; HBeAg = Hepatitis B e antigen; Anti-HCV = antibody to hepatitis C virus; IFN = interferon

#### Cirrhosis

Coinfected patients have higher rates of cirrhosis with decompensation. One cross-sectional study found higher rates of cirrhosis (44% vs. 21%) and decompensated liver disease (24% vs. 6%) in coinfected patients compared to patients with chronic HBV monoinfection [16]. Moreover, HBV replication in coinfected patients (detectable serum HBV DNA) has been correlated with higher rates of cirrhosis, Knodell score, piecemeal necrosis and fibrosis [29]. A Saudi Arabian study found that coinfected patients (compared to HCV monoinfected patients) had more decompensated liver disease with a higher proportion of cirrhosis (95% vs. 48.5%) and Child-Pugh class C (37% vs. 0%) [42].

#### Hepatocellular Carcinoma

Coinfection with HBV and HCV has been shown in many case-control studies to correlate with an increased risk of developing HCC [4,42]. One study found a rate of HCC in coinfected patients of 63% compared to 15% in HCV monoinfection [42]. Benvegnu et al. [1] conducted a prospective study of 290 cirrhotic patients and found that coinfection (detectable anti-HCV and HBsAg) was an independent predictor for development of HCC in a univariate and multivariate analysis (p < 0.02, p < 0.05 respectively). A subsequent longitudinal study confirmed these results, and reported a rate of incidence of HCC (per 100 person years) of 6.4 in dually infected patients, compared to 2.0 in HBV and 3.7 in HCV monoinfected patients, and a 45% cumulative risk of developing HCC at 10 years in coinfected patients, compared with 16% and 28% in HBV and HCV monoinfected controls [2]. Similarly, A South African study reported an 83-fold increased for developing HCC among coinfected patients compared to patients without hepatitis B or C [43]. Because of the likely increased risk of developing HCC, coinfected patients should receive regular 6-month and possible more frequent screening with ultrasound of the liver and serum alfa-fetoprotein levels.

### Treatment

#### General Principles

Well established treatment guidelines exist for patients with chronic hepatitis B and chronic hepatitis C [44-49]. In regards to HBV infection, the Asian-Pacific Association for the Study of the Liver (APASL), the European Association for the Study of the Liver (EASL) and the American Association for the Study of Liver Diseases (AASLD) recommend treating patients who have moderate to severe chronic hepatitis as evidenced by >2-fold elevation of ALT levels or significant findings on liver biopsy associated with HBV DNA >10^5 ^copies/mL. Patients may have detectable HBeAg associated with wild type virus or HBeAg-negative from infection with the precore or core promoter mutant virus. Currently licensed drugs for chronic hepatitis B in the United States include interferon alfa-2b, lamivudine, adefovir, entecavir, and peginterferon alfa-2a. Patients with fulminant hepatitis and decompensated cirrhosis are not likely to respond to antiviral agents and are candidates for liver transplantation (Table 1). Patients with chronic hepatitis C with detectable serum HCV RNA are candidates for 24 to 48 weeks of antiviral therapy based on genotype. Currently standard treatment for hepatitis C is peginterferon alfa-2a or 2b plus ribavirin. As with chronic hepatitis B, patients with decompensated liver disease or fulminant hepatitis (rare with acute hepatitis C) are candidates for liver transplantation (Table 2).

**Table 1:** AASLD Recommendations for Treatment of Chronic Hepatitis B

**HBeAg**	**HBV DNA**	**ALT**	**Treatment Strategy**
+	+	≤2 × ULN	Low efficacy with current treatment. Observe; consider treatment when ALT becomes elevated
+	+	>2 × ULN	IFN-α, LAM, or ADV may be used as initial therapy End point of treatment = seroconversion from HbeAg to anti-Hbe Duration of therapy: • IFN-α: 16 weeks • LAM: minimum 1 year, continue for 3–6 months after HbeAg seroconversion • ADV: minimum 1 year IFN-α nonresponders/contraindications to IFN-α → LAM or ADV LAM resistance → ADV
-	+	>2 × ULN	IFN-α, LAM or ADV may be used as initial therapy, IFN-α or ADV is preferred End point of treatment = sustained normalization of ALT and undetectable HBV DNA by PCR assay Duration of therapy: • IFN-α: 1 year • LAM: > 1 year • ADV: > 1 year IFN-α nonresponders/contraindications to IFN-α → LAM or ADV LAM resistance → ADV
-	-	≤2 × ULN	No treatment required
±	+	Cirrhosis	Compensated: LAM or ADV Decompensated: LAM (or ADV); Refer for liver transplant. IFN-α contraindicated.
±	-	Cirrhosis	Compensated: Observe Decompensated: Refer for liver transplant

*HBV DNA > 10^5^. Abbreviations: HBeAg: hepatitis B e antigen; HBV: hepatitis B virus; ALT: alanine aminotransferase; ULN: upper limit of normal; IFN-α: interferon alfa; LAM: lamivudine; ADV: adefovir; PCR: polymerase chain reaction. Source: Lok AS, McMahon BJ. Chronic hepatitis B: update of recommendations. Hepatology 2004;39:857-61.

**Table 2:** AASLD Recommendations for Treatment of Chronic Hepatitis C

**Therapy Widely Accepted**	**Therapy Contraindicated**	**Treatment Recommendations**
• Detectable HCV RNA • 18 years of age or older • Elevated ALT • Liver biopsy showing chronic hepatitis with significant fibrosis • Compensated liver disease (total serum bilirubin <1.5 g/dL; INR <1.5; albumin >3.4 g/dL; platelet count >75,000 k/mm^3^; and no evidence of hepatic encephalopathy or ascites) • Acceptable hematological and biochemical indices (hemoglobin >13 g/dL for men and >12 g/dL for women; neutrophil count >1.5 k/mm^3^; creatinine <1.5 mg/dL) • Not treated previously for HCV infection • History of depression but well controlled • Patient willing to be treated and to conform to treatment requirements	• Major, uncontrolled depression • Renal, heart, or lung transplant recipient • Autoimmune hepatitis or other condition known to be exacerbated by interferon and ribavirin • Untreated hyperthyroidism • Pregnant or unwilling/unable to comply with adequate contraception • Severe concurrent disease such as severe hypertension, heart failure, significant coronary artery disease, poorly controlled diabetes, obstructive pulmonary disease • Under 3 years of age • Known hypersensitivity to drugs used to treat HCV	Genotype 1 HCV infection: • Peginterferon plus ribavirin (1000–1200 mg daily) for 48 weeks • Treatment may be discontinued in patients who do not achieve an EVR at 12 weeks • In patients who have negative HCV RNA at 48 weeks, retest HCV RNA at 72 weeks to confirm SVR Genotype 2 or Genotype 3 infection: • Peginterferon plus ribavirin (800 mg daily) for 24 weeks • In patients who have negative HCV RNA at 24 weeks, retest HCV RNA at 48 weeks to confirm SVR

Abbreviations: INR: international normalized ratio; EVR: early virologic response; SVR: sustained virologic response. Source: Strader DB, Wright T, Thomas DL, Seeff LB. Diagnosis, management, and treatment of hepatitis C. Hepatology 2004;39:1147-71.

There is no currently established standard of care for patients who are coinfected with HBV and HCV. In general, the same treatment criteria should be applied to patients who are HBC/HCV dually infected as are applied to monoinfected patients (Tables 1 and 2). Initiation of treatment, as with both HBV and HCV, is recommended in patients with active chronic hepatitis or cirrhosis prior to decompensation (Figure 1). Given the complex interaction of HBV and HCV both with each other and with the immune system, care must be taken to select the most appropriate antiviral regimen based on serologic markers and levels of viremia. Because of its activity against both viruses, interferon therapy has been the most studied. The published data on the antiviral treatment of HBV and HCV coinfection is reviewed in the following discussion and summarized in Table 4.

**Table 4:** Medication Trials in Hepatitis B and Hepatitis C Coinfected Patients

**Author [Ref]**	**Patients**	**#**	**Treatment × Duration**	**HCV SVR**	**HBV DNA negative**	**HBsAg loss**	**HBeAg loss**	**SBR**
**Interferon Trials**								

Gehenot [53]	Anti-HCV+ HCV RNA+ HBsAg+ HBV DNA-	16	IFNα 3 MU TIW × 6 mo	N/A	N/A	12.5%	N/A	19%
Weltman [3]	Anti-HCV+ HBsAg+	8	IFNα 3 MU TIW × 6 mo	N/A	N/A	12.5%	N/A	12.5%
Guptan [54]	Anti-HCV+ HCV RNA+ HBsAg+ HBV DNA+	7	IFNα 6 MU TIW × 6 mo	29%	86%	28.6%	100%	0%
Villa [55]	Anti-HCV+ HCV RNA+ HBsAg+	30	IFNα 9 MU TIW × 6 mo or 6 MU TIW × 6 mo	16.7% (31%)*	66.7% (100%)*	3%	N/A	20% (37.5%)*
Utili [23]	Anti-HCV+ HBeAg ± HCV RNA ±	16	IFNα 5 MU TIW × 12 mo	43.8%	N/A	N/A	15.4%	50%
Zignego [10]	Anti-HCV+ HBV DNA+ HBsAg-	14	IFNα 3 MU TIW × 12 mo	0%	0%	N/A	N/A	0%
Liaw [56]	Anti-HCV+ HBV DNA+ HBeAg+	15	IFNα 9 MU TIW × 14 wk or 4–6 MU TIW × 12 wk	0%	6.7%	6.7%	6.7%	6.7%

**Interferon plus ribavirin trials**								

Liu [57]	Anti-HCV+ HCV RNA+ HBsAg+	21	IFNα 6 MU TIW × 3 mo + 3 MU TIW × 3 mo + ribavirin × 6 mo	43%	35%	0%	100%	43%
Hung [58]	Anti-HCV+ HCV RNA+ HBsAg+	36	IFNα 3–5 MU TIW + ribavirin × 6 mo	69%	11%	0%	0%	56%
Chuang [59]	Anti-HCV+ HCV RNA+ HBsAg+	42	IFNα 6 MU TIW + ribavirin × 6 mo	69%	31.3%	14.3%	50%	54.8%

**Interferon plus lamivudine trials**								

Marrone [61]	HBeAg+ HBV DNA+ HCV RNA+	8	IFN 5 MU TIW × 12 mo + lamivudine × 18 mo	50%	37.5%	0%	37.5%	50%

*9 MU arm. Abbreviations: # = number of patients; Ref = reference; SVR = sustained virologic response; SBR = sustained biochemical response; HCV = hepatitis C virus; HBV = hepatitis B virus; Anti-HCV = Antibody to hepatitis C virus; HBsAg = hepatitis B surface antigen; HBeAg = hepatitis B e antigen; mo = month; wk = week; TIW = thrice weekly; IFNα = interferon alfa

#### Interferon

Interferon is an immunomodulating medication with antiviral and antiproliferative effects and has been well studied in patients with chronic viral hepatitis. Interferon alpha (IFNα) is an approved treatment for both chronic hepatitis B and C. In appropriately selected patients with hepatitis C, interferon treatment led to sustained virological response (SVR), i.e. negative HCV RNA 6 months following completion of treatment, in approximately 10% of HCV patients. These results are improved when IFN is used with ribavirin (SVR up to 43%), and when peginterferon plus ribavirin is used (SVR up to 56%) [45]. In chronic HBV infection, IFN is indicated for patients with chronic hepatitis with elevated ALT and HBV DNA levels, and has the benefits of a lack of resistance and a durable response in those who respond to therapy. In studies of HBV patients, interferon is effective in roughly 35% of patients [50]. Peginterferon has also recently shown promise in treatment of hepatitis B [51], and peginterferon alfa-2a was recently licensed in the United States.

IFN has been the most studied agent in treatment of coinfected patients because of the wealth of experience with this agent in viral hepatitis and its proven activity against both viruses. One of the first case reports of successful treatment with IFN of a coinfected patient (resulting in HBeAg loss and a SVR of HCV infection) was published by Burt et al. in 1993 [52]. Several case series were subsequently reported. In 1995, Géhénot et al. [53] studied 16 patients with histologically proven chronic hepatitis with positive anti-HCV, HCV RNA, HBsAg, and anti-HBe (HBV DNA negative), compared to patients with chronic HCV infection alone. Treatment consisted of IFNα at 3 million units (MU) thrice weekly (TIW) for 6 months. A similar rate of sustained biochemical response (normal ALT at 6 months following treatment; SBR) was achieved in coinfected patients compared to controls (19 vs. 21%). Two patients treated with IFN seroconverted to HBsAg negative. This study demonstrated that comparable results could be obtained with interferon in coinfected patients who do not have evidence of HBV replication. A subsequent study by Weltman et al. [3] of 8 coinfected patients treated with the same regimen (IFN alfa-2b 3 MU TIW for 6 months) reported similar results, with 1 patient experiencing SBR (12.5%). The patient with SBR seroconverted at 6 months to HBsAg negative/anti-HBs positive. An Indian study of 7 dually infected patients (HBsAg, HBV DNA, anti-HCV, HCV RNA positive) used a higher dose of IFN alfa-2b (6 MU) for 6 months [54]. After 6 months follow-up, the authors reported 100% of patients lost HBV DNA, 100% of HBeAg-positive patients lost HBeAg (3/3), and 29% lost HCV RNA (i.e. SVR = 29%). Utili et al. [23] studied a cohort of 32 HBV/HCV coinfected patients, 16 of whom received IFN treatment (5 MU TIW for 12 months). They report an overall SVR rate of 43.8% for HCV infection, and this rate was increased in patients who were HBeAg negative (66.7%). Loss of HBeAg occurred in 2 of 13 patients (15.4%).

Villa et al. [55] conducted a larger prospective randomized trial of 30 patients with HBV/HCV coinfection (HBsAg-positive, Anti-HCV-positive, HCV RNA-positive), in which patients received either 6 or 9 MU IFN-α TIW for 6 months. This study found that higher dose IFN was more effective in inducing clearance of HCV RNA (31.2% vs. 0%, p = 0.045) and HBV DNA (100% vs. 0%), as well as inducing a SBR (37.5% vs. 0%, p = 0.019) compared to the lower dose. Histological scores were better in patients in the high-dose arm as well. This was the first study to suggest that higher doses of IFN may be indicated in coinfected patients.

Several studies have reported detectable HBV DNA in patients with chronic hepatitis C but negative HBsAg. This so-called "serologically silent" HBV infection or "inapparent coinfection" has been correlated with impaired response to IFN treatment. Zignego et al. [10] reported significantly worsened results in 14 chronically infected HCV patients with inapparent HBV coinfection (anti-HCV-positive, HBV DNA-positive, HBsAg-negative). Patients were treated with IFN alfa-2a TIW for 12 months. Four out of 14 patients had normal ALT levels at the end of therapy (28%), but all had relapsed within 6 month post-treatment, and thus none had a SVR. Fukuda et al. [14] also found that silent HBV infection was associated with higher ALT levels, greater histological activity scores and poor efficacy of IFN treatment. Some have proposed that the impaired response to IFN in such patients may be due to HBV-mediated down-regulation of intrahepatic IFN receptor gene expression [15].

A hepatitis C flare has been described in a coinfected patient who had HBeAg/HBV DNA clearance in response to IFN [56]. For this reason, some investigators have raised concern that IFN treatment of coinfected patients carries the risk of a severe hepatitis flare if the suppressive effect of one virus is removed, thereby allowing the other virus to become active.

Though there have been no published studies of the use of peginterferon (peg-IFN) in coinfected patients, the use of this agent will likely replace standard interferon in treatment of HBV/HCV coinfection, as peginterferon is now the standard of care for the treatment of chronic hepatitis C, and has recently been approved for the treatment of chronic hepatitis B.

#### Interferon plus Ribavirin

Several groups have published studies addressing treatment of coinfected patients with antiviral combination therapy with IFNα plus ribavirin. Liu et al. [57] treated 24 dually infected patients (HBsAg positive/anti-HCV positive) with IFN alfa-2a (6 MU TIW for 12 weeks followed by 3 MU TIW for 12 weeks), concurrently with ribavirin 1200 mg daily for 24 weeks. Seventeen patients were positive for both HBV DNA and HCV RNA. Results showed a SVR rate of 43% for clearance of HCV RNA, compared to 60% in similarly treated HCV monoinfected controls. Six of 17 patients with detectable HBV DNA at baseline had disappearance of HBV DNA at the end of treatment persisting to 24 weeks post-treatment (35%). A SBR rate of 43% was reported. These results demonstrate the effectiveness of combined IFN and ribavirin in coinfected patients, with a rate of SVR and SBR comparable to HCV monoinfected patients.

Hung et al. [58] treated 36 patients with HBV/HCV coinfection (HBsAg-positive/anti-HCV-positive/HCV RNA-positive) with combination IFN alfa-2b (3 or 5 MU TIW) and ribavirin (800–1200 mg/day) for 24 weeks. This study reported a SVR rate of 69% and SBR rate of 56%. Loss of HBV DNA was found in 2 of 18 patients with HBV DNA detected at baseline (11%). Interestingly, 53% of patients with negative HBV DNA at baseline had reactivation of HBV DNA at the 48 weeks of follow-up.

Chuang et al. [59] studied combination therapy with high-dose IFN alfa-2b (6 MU 3 times weekly for 24 weeks) and ribavirin (1000–1200 mg daily for 24 weeks) in 42 coinfected patients, compared to a control group of HCV monoinfected patients. The investigators found comparable rates of SVR in HCV coinfected (69.0%) and monoinfected (67.2%) patients (IFN naïve). Disappearance of HBV DNA occurred in 5 of 16 (31%) patients with positive HBV DNA at baseline. Viral interaction was evident, in that coinfected patients who achieved a SVR (compared HCV nonresponders) were less likely to achieve HBV DNA clearance (8.3% vs. 100%), and more likely to have reactivation of HBV (58.8% vs. 12.5%) or HBV flares (44.8% vs. 8.3%). Of the patients who cleared HBV DNA, 4 of 5 were patients who did not achieve a SVR with undetectable HCV RNA following combination therapy. Nonetheless, the high SVR rate for HCV infection in this population provides further evidence that IFN plus ribavirin combination therapy can be effective in coinfected patients, especially those who have active HCV replication.

As described in the above studies, successful treatment of chronic HCV infection may correlate to HBV reactivation and flaring. Yalcin et al. reported a severe hepatitis B flare in a patient with HBV/HCV coinfection (HBV DNA-negative) undergoing treatment with IFN and ribavirin [60]. This patient's hepatitis improved after discontinuation of therapy, but a relapse of HCV infection with rapid progression to cirrhosis occurred thereafter. Clinicians must exercise caution when treating coinfected patients with combination IFN plus ribavirin given this risk of HBV reactivation.

#### Interferon plus Lamivudine

One study of lamivudine therapy in addition to IFN for coinfected patients has been published by Marrone et al [61]. Eight patients with dually active HBV and HCV (HBeAg-positive/HBV DNA-positive/HCV RNA-positive) were treated with 5 MU of IFN and lamivudine 100 mg/day for 12 months followed by lamivudine alone for 6 months. Three patients had clearance of HBeAg, 3 had clearance of HBV DNA (37.5%), and post-treatment ALT levels normalized in 4 of 8 (50%) treated patients. Four patients (50%) also had clearance of HCV RNA that was persistent at 12 months post-treatment (i.e. HCV SVR = 50%). This initial study suggests that the addition of lamivudine to IFN may be effective in coinfected patients with chronic hepatitis C and active HBV replication. Further studies on this combination therapy in larger groups of patients must be performed in order to confirm these results.

#### Adefovir and Entecavir

There have been no published studies regarding treatment of coinfected patients with the newer agents adefovir and entecavir. However, these agents may be useful, particularly in patients with HBV-dominant disease. Studies need to be performed using these agents before they can be recommended for routine usage.

#### Transplantation

The United Network of Organ Sharing (UNOS) reported that 14 patients were transplanted for combined hepatitis B and C in the United States in 2004, and 434 patients have been transplanted for this indication since 1988 [62]. There are limited data on the post-transplant course of such patients. One small study by Huang et al. [63] reported results of 19 patients with coinfection (either acquired or persistent) following transplantation. This study found that survival of patients with HBV monoinfection was worse than that of dually infected patients, suggesting a possible beneficial role of HCV in the immunosuppressed post-transplant population. Further studies are needed to clarify the clinical course and optimal management of coinfected post-transplant patients.

### Special Populations

#### Triple Infection with HBV, HCV and Hepatitis Delta Virus

Hepatitis delta virus (HDV) infects only patients with preexisting HBV infection. Triple hepatotropic viral infections with HBV, HCV and HDV can result in more severe hepatitis, and therefore compel the clinician to offer treatment [8]. Few studies have been published regarding treatment of patients with triple hepatitis virus infection. Weltman et al. [3] studied 7 patients with triple infection who received IFN therapy. One patient had a reported SBR, and 2 patients were withdrawn from treatment due to side effects. Interferon treatment is a reasonable recommendation despite the paucity of data to support its use. Further studies are needed on such patients to determine optimal therapy.

#### Triple Infection with HBV, HCV and Human Immunodeficiency Virus

Triple infection with HBV, HCV and the human immunodeficiency virus (HIV) is a complex clinical scenario, due to the interaction of HBV and HCV, and the impact of HIV on the immune system. In addition, a majority of patients with HIV/HCV coinfection are infected with HCV genotype 1, decreasing their response to interferon therapy and thereby rendering treatment more difficult [64]. Furthermore, HCV/HIV coinfection has been shown to result in more severe liver disease and an increased risk of liver disease-related death [65]. No standard of care exists for such patients, and treatment must be individualized and coordinated with an HIV specialist. Infection of HIV must be controlled before treatment of viral hepatitis can be considered [66]. Few studies have been performed on treatment of patients with triple infection, so treatment algorithms are often extrapolated from results of trials of patients with either HBV/HIV or HCV/HIV coinfection. IFN alone is associated with a SVR rate of approximately 17% in HCV/HIV coinfected patients, and this rate increases to 25% with the addition of ribavirin [67]. Lamivudine has been used in HBV/HIV coinfected patients, but is associated with a high rate of resistance [68]. There is promise for the use of newer agents such as adefovir, entecavir, tenofovir, and emtricitabine, but no studies have corroborated their utility in this population [66].

### Summary of Treatment Recommendations

Thorough serologic and virologic testing is required in dually infected patients prior to consideration of therapy. Assessment of the "dominant" virus is helpful in determining a treatment strategy. Caution must be taken with treatment of coinfected individuals, as exacerbations of liver disease after initiation of therapy have been described, likely due to loss of viral suppression from the successfully treated dominant virus. Patients are candidates for therapy if they meet the inclusion criteria for standard treatment guidelines for either HBV or HCV monoinfection (Tables 1 and 2). In coinfected patients with HCV dominant disease, IFN plus ribavirin treatment has been well studied and has proven efficacy. In patients with HBV dominant disease, IFN with or without lamivudine is a reasonable option. Further studies of other HBV treatment agents such as adefovir and entecavir are needed before these agents can be routinely recommended, though they may be used on a case by case basis. In addition, future studies are needed to assess the effectiveness of peginterferon as well as triple therapy with lamivudine, IFN, and ribavirin in coinfected patients, though peginterferon should generally be used in place of standard interferon in coinfected patients given its proven efficacy in HBV and HCV monoinfected patients. Referral to a transplant center is indicated for patients with decompensated cirrhosis, fulminant hepatitis, or HCC in appropriate patients. In patients with triple HCV/HBV/HDV infections, few treatment studies have been published, but IFN is a reasonable treatment. Patients who have triple infection with HIV/HBV/HCV should have their care coordinated with an HIV specialist.

## Conclusion

Coinfection with HBV and HCV is not uncommon, especially within areas of high prevalence of hepatitis B. Dual infections present unique management challenges given the complex interaction of HBV and HCV, and the propensity for developing more severe liver disease. Treatment options mostly include IFN with or without lamivudine or ribavirin. Treatment decisions should be made based upon the determination of the "dominant" hepatitis virus. Caution must be exercised in treating coinfected patients, as flares of the untreated virus may occur. No standard of care has been established for treatment of coinfected patients, and larger randomized, controlled trials are needed to clarify the optimal treatment for such patients and the role of newer antiviral agents.

## References

[B1] Benvegnu L, Fattovich G, Noventa F, Tremolada F, Chemello L, Cecchetto A, Alberti A (1994). Concurrent hepatitis B and C virus infection and risk of hepatocellular carcinoma in cirrhosis. A prospective study. Cancer.

[B2] Chiaramonte M, Stroffolini T, Vian A, Stazi MA, Floreani A, Lorenzoni U, Lobello S, Farinati F, Naccarato R (1999). Rate of incidence of hepatocellular carcinoma in patients with compensated viral cirrhosis. Cancer.

[B3] Weltman MD, Brotodihardjo A, Crewe EB, Farrell GC, Bilous M, Grierson JM, Liddle C (1995). Coinfection with hepatitis B and C or B, C and delta viruses results in severe chronic liver disease and responds poorly to interferon-alpha treatment. J Viral Hepat.

[B4] Kaklamani E, Trichopoulos D, Tzonou A, Zavitsanos X, Koumantaki Y, Hatzakis A, Hsieh CC, Hatziyannis S (1991). Hepatitis B and C viruses and their interaction in the origin of hepatocellular carcinoma. JAMA.

[B5] Lee WM (1997). Hepatitis B virus infection. N Engl J Med.

[B6] (1999). Hepatitis C–global prevalence (update). Wkly Epidemiol Rec.

[B7] Atanasova MV, Haydouchka IA, Zlatev SP, Stoilova YD, Iliev YT, Mateva NG (2004). Prevalence of antibodies against hepatitis C virus and hepatitis B coinfection in healthy population in Bulgaria. A seroepidemiological study. Minerva Gastroenterol Dietol.

[B8] Liaw YF (1995). Role of hepatitis C virus in dual and triple hepatitis virus infection. Hepatology.

[B9] Gaeta GB, Stornaiuolo G, Precone DF, Lobello S, Chiaramonte M, Stroffolini T, Colucci G, Rizzetto M (2003). Epidemiological and clinical burden of chronic hepatitis B virus/hepatitis C virus infection. A multicenter Italian study. J Hepatol.

[B10] Zignego AL, Fontana R, Puliti S, Barbagli S, Monti M, Careccia G, Giannelli F, Giannini C, Buzzelli G, Brunetto MR (1997). Relevance of inapparent coinfection by hepatitis B virus in alpha interferon-treated patients with hepatitis C virus chronic hepatitis. J Med Virol.

[B11] Liaw YF (2002). Hepatitis C virus superinfection in patients with chronic hepatitis B virus infection. J Gastroenterol.

[B12] Liaw YF, Yeh CT, Tsai SL (2000). Impact of acute hepatitis B virus superinfection on chronic hepatitis C virus infection. Am J Gastroenterol.

[B13] Liaw YF, Chen YC, Sheen IS, Chien RN, Yeh CT, Chu CM (2004). Impact of acute hepatitis C virus superinfection in patients with chronic hepatitis B virus infection. Gastroenterology.

[B14] Fukuda R, Ishimura N, Niigaki M, Hamamoto S, Satoh S, Tanaka S, Kushiyama Y, Uchida Y, Ihihara S, Akagi S (1999). Serologically silent hepatitis B virus coinfection in patients with hepatitis C virus-associated chronic liver disease: clinical and virological significance. J Med Virol.

[B15] Fukuda R, Ishimura N, Hamamoto S, Moritani M, Uchida Y, Ishihara S, Akagi S, Watanabe M, Kinoshita Y (2001). Co-infection by serologically-silent hepatitis B virus may contribute to poor interferon response in patients with chronic hepatitis C by down-regulation of type-I interferon receptor gene expression in the liver. J Med Virol.

[B16] Fong TL, Di Bisceglie AM, Waggoner JG, Banks SM, Hoofnagle JH (1991). The significance of antibody to hepatitis C virus in patients with chronic hepatitis B. Hepatology.

[B17] Chu CM, Yeh CT, Liaw YF (1998). Low-level viremia and intracellular expression of hepatitis B surface antigen (HBsAg) in HBsAg carriers with concurrent hepatitis C virus infection. J Clin Microbiol.

[B18] Crespo J, Lozano JL, de la Cruz F, Rodrigo L, Rodriguez M, San Miguel G, Artinano E, Pons-Romero F (1994). Prevalence and significance of hepatitis C viremia in chronic active hepatitis B. Am J Gastroenterol.

[B19] Liaw YF, Tsai SL, Chang JJ, Sheen IS, Chien RN, Lin DY, Chu CM (1994). Displacement of hepatitis B virus by hepatitis C virus as the cause of continuing chronic hepatitis. Gastroenterology.

[B20] Liaw YF, Lin SM, Sheen IS, Chu CM (1991). Acute hepatitis C virus superinfection followed by spontaneous HBeAg seroconversion and HBsAg elimination. Infection.

[B21] Dai CY, Yu ML, Chuang WL, Lin ZY, Chen SC, Hsieh MY, Wang LY, Tsai JF, Chang WY (2001). Influence of hepatitis C virus on the profiles of patients with chronic hepatitis B virus infection. J Gastroenterol Hepatol.

[B22] Sheen IS, Liaw YF, Lin DY, Chu CM (1994). Role of hepatitis C and delta viruses in the termination of chronic hepatitis B surface antigen carrier state: a multivariate analysis in a longitudinal follow-up study. J Infect Dis.

[B23] Utili R, Zampino R, Bellopede P, Marracino M, Ragone E, Adinolfi LE, Ruggiero G, Capasso M, Indolfi P, Casale F (1999). Dual or single hepatitis B and C virus infections in childhood cancer survivors: long-term follow-up and effect of interferon treatment. Blood.

[B24] Shih CM, Lo SJ, Miyamura T, Chen SY, Lee YH (1993). Suppression of hepatitis B virus expression and replication by hepatitis C virus core protein in HuH-7 cells. J Virol.

[B25] Schuttler CG, Fiedler N, Schmidt K, Repp R, Gerlich WH, Schaefer S (2002). Suppression of hepatitis B virus enhancer 1 and 2 by hepatitis C virus core protein. J Hepatol.

[B26] Pontisso P, Gerotto M, Ruvoletto MG, Fattovich G, Chemello L, Tisminetzky S, Baralle F, Alberti A (1996). Hepatitis C genotypes in patients with dual hepatitis B and C virus infection. J Med Virol.

[B27] Sato S, Fujiyama S, Tanaka M, Yamasaki K, Kuramoto I, Kawano S, Sato T, Mizuno K, Nonaka S (1994). Coinfection of hepatitis C virus in patients with chronic hepatitis B infection. J Hepatol.

[B28] Pontisso P, Ruvoletto MG, Fattovich G, Chemello L, Gallorini A, Ruol A, Alberti A (1993). Clinical and virological profiles in patients with multiple hepatitis virus infections. Gastroenterology.

[B29] Zarski JP, Bohn B, Bastie A, Pawlotsky JM, Baud M, Bost-Bezeaux F, Tran van Nhieu J, Seigneurin JM, Buffet C, Dhumeaux D (1998). Characteristics of patients with dual infection by hepatitis B and C viruses. J Hepatol.

[B30] Ohkawa K, Hayashi N, Yuki N, Masuzawa M, Kato M, Yamamoto K, Hosotsubo H, Deguchi M, Katayama K, Kasahara A (1995). Long-term follow-up of hepatitis B virus and hepatitis C virus replicative levels in chronic hepatitis patients coinfected with both viruses. J Med Virol.

[B31] Doi T, Yamada G, Endo H, Nishimoto H, Takahashi M, Miyamoto R, Fujiki S, Shimomura H, Mizuno M, Tsuji T (1992). Hepatitis type C virus infection in patients with type B chronic liver disease. Gastroenterol Jpn.

[B32] Jardi R, Rodriguez F, Buti M, Costa X, Cotrina M, Galimany R, Esteban R, Guardia J (2001). Role of hepatitis B, C, and D viruses in dual and triple infection: influence of viral genotypes and hepatitis B precore and basal core promoter mutations on viral replicative interference. Hepatology.

[B33] Liaw YF (2001). Concurrent hepatitis B and C virus infection: Is hepatitis C virus stronger?. J Gastroenterol Hepatol.

[B34] Liaw YF, Chu CM, Chang-Chien CS, Wui CS (1982). Simultaneous acute infections with hepatitis non-A, non-B, and B viruses. Dig Dis Sci.

[B35] Baginski I, Chemin I, Hantz O, Pichoud C, Jullien AM, Chevre JC, Li JS, Vitvitski L, Sninsky JJ, Trepo C (1992). Transmission of serologically silent hepatitis B virus along with hepatitis C virus in two cases of posttransfusion hepatitis. Transfusion.

[B36] Mimms LT, Mosley JW, Hollinger FB, Aach RD, Stevens CE, Cunningham M, Vallari DV, Barbosa LH, Nemo GJ (1993). Effect of concurrent acute infection with hepatitis C virus on acute hepatitis B virus infection. Bmj.

[B37] Sagnelli E, Coppola N, Scolastico C, Mogavero AR, Filippini P, Piccinino F (2001). HCV genotype and "silent" HBV coinfection: two main risk factors for a more severe liver disease. J Med Virol.

[B38] Cacciola I, Pollicino T, Squadrito G, Cerenzia G, Orlando ME, Raimondo G (1999). Occult hepatitis B virus infection in patients with chronic hepatitis C liver disease. N Engl J Med.

[B39] Chu CM, Sheen IS, Liaw YF (1994). The role of hepatitis C virus in fulminant viral hepatitis in an area with endemic hepatitis A and B. Gastroenterology.

[B40] Feray C, Gigou M, Samuel D, Reyes G, Bernuau J, Reynes M, Bismuth H, Brechot C (1993). Hepatitis C virus RNA and hepatitis B virus DNA in serum and liver of patients with fulminant hepatitis. Gastroenterology.

[B41] Wu JC, Chen CL, Hou MC, Chen TZ, Lee SD, Lo KJ (1994). Multiple viral infection as the most common cause of fulminant and subfulminant viral hepatitis in an area endemic for hepatitis B: application and limitations of the polymerase chain reaction. Hepatology.

[B42] Mohamed Ael S, al Karawi MA, Mesa GA (1997). Dual infection with hepatitis C and B viruses: clinical and histological study in Saudi patients. Hepatogastroenterology.

[B43] Kew MC, Yu MC, Kedda MA, Coppin A, Sarkin A, Hodkinson J (1997). The relative roles of hepatitis B and C viruses in the etiology of hepatocellular carcinoma in southern African blacks. Gastroenterology.

[B44] Lok AS, McMahon BJ (2004). Chronic hepatitis B: update of recommendations. Hepatology.

[B45] Strader DB, Wright T, Thomas DL, Seeff LB (2004). Diagnosis, management, and treatment of hepatitis C. Hepatology.

[B46] (1999). EASL International Consensus Conference on Hepatitis C. Paris, 26–28, February Consensus Statement. European Association for the Study of the Liver. J Hepatol.

[B47] de Franchis R, Hadengue A, Lau G, Lavanchy D, Lok A, McIntyre N, Mele A, Paumgartner G, Pietrangelo A, Rodes J (2003). EASL International Consensus Conference on Hepatitis B. 13–14 September, 2002 Geneva, Switzerland. Consensus statement (long version). J Hepatol.

[B48] Liaw YF, Leung N, Guan R, Lau GK, Merican I, McCaughan G, Gane E, Kao JH, Omata M (2005). Asian-Pacific consensus statement on the management of chronic hepatitis B: a 2005 update. Liver Int.

[B49] Keeffe EB, Dieterich DT, Han SH, Jacobson IM, Martin P, Schiff ER, Tobias H, Wright TL (2004). A treatment algorithm for the management of chronic hepatitis B virus infection in the United States. Clin Gastroenterol Hepatol.

[B50] Wong DK, Cheung AM, O'Rourke K, Naylor CD, Detsky AS, Heathcote J (1993). Effect of alpha-interferon treatment in patients with hepatitis B e antigen-positive chronic hepatitis B. A meta-analysis. Ann Intern Med.

[B51] Chan HL, Leung NW, Hui AY, Wong VW, Liew CT, Chim AM, Chan FK, Hung LC, Lee YT, Tam JS (2005). A randomized, controlled trial of combination therapy for chronic hepatitis B: comparing pegylated interferon-alpha2b and lamivudine with lamivudine alone. Ann Intern Med.

[B52] Burt M, Chapman BA, Scrimshaw BJ, Jennings LC, George PM (1993). Concurrent hepatitis B and C infection treated successfully with alpha interferon. Aust NZ J Med.

[B53] Gehenot M, Marcellin P, Colin JF, Martinot M, Benhamou JP, Erlinger S (1995). Alpha Interferon Therapy in HBsAg Positive Patients with Chronic Hepatitis C. Hepatology.

[B54] Guptan RC, Thakur V, Raina V, Sarin SK (1999). Alpha-interferon therapy in chronic hepatitis due to active dual infection with hepatitis B and C viruses. J Gastroenterol Hepatol.

[B55] Villa E, Grottola A, Buttafoco P, Colantoni A, Bagni A, Ferretti I, Cremonini C, Bertani H, Manenti F (2001). High doses of alpha-interferon are required in chronic hepatitis due to coinfection with hepatitis B virus and hepatitis C virus: long term results of a prospective randomized trial. Am J Gastroenterol.

[B56] Liaw YF, Chien RN, Lin SM, Yeh CT, Tsai SL, Sheen IS, Chu CM (1997). Response of patients with dual hepatitis B virus and C virus infection to interferon therapy. J Interferon Cytokine Res.

[B57] Liu CJ, Chen PJ, Lai MY, Kao JH, Jeng YM, Chen DS (2003). Ribavirin and interferon is effective for hepatitis C virus clearance in hepatitis B and C dually infected patients. Hepatology.

[B58] Hung CH, Lee CM, Lu SN, Wang JH, Tung HD, Chen CH, Changchien CS (2005). Combination therapy with interferon-alpha and ribavirin in patients with dual hepatitis B and hepatitis C virus infection. J Gastroenterol Hepatol.

[B59] Chuang WL, Dai CY, Chang WY, Lee LP, Lin ZY, Chen SC, Hsieh MY, Wang LY, Yu ML (2005). Viral interaction and responses in chronic hepatitis C and B coinfected patients with interferon-alpha plus ribavirin combination therapy. Antivir Ther.

[B60] Yalcin K, Degertekin H, Yildiz F, Kilinc N (2003). A severe hepatitis flare in an HBV-HCV coinfected patient during combination therapy with alpha-interferon and ribavirin. J Gastroenterol.

[B61] Marrone A, Zampino R, D'Onofrio M, Ricciotti R, Ruggiero G, Utili R (2004). Combined interferon plus lamivudine treatment in young patients with dual HBV (HBeAg positive) and HCV chronic infection. J Hepatol.

[B62] United Network of Organ Sharing.

[B63] Huang EJ, Wright TL, Lake JR, Combs C, Ferrell LD (1996). Hepatitis B and C coinfections and persistent hepatitis B infections: clinical outcome and liver pathology after transplantation. Hepatology.

[B64] Rubio Caballero M, Rubio Rivas C, Nogues Biau A, Manonelles Fernandez A (2005). [Epidemiology of chronic hepatitis C virus in patients infected by human immunodeficiency virus. Study of 767 patients]. Med Clin (Barc).

[B65] Soriano V, Martin-Carbonero L, Garcia-Samaniego J, Puoti M (2001). Mortality due to chronic viral liver disease among patients infected with human immunodeficiency virus. Clin Infect Dis.

[B66] Sterling RK (2003). Triple infection with human immunodeficiency virus, hepatitis C virus, and hepatitis B virus: a clinical challenge. Am J Gastroenterol.

[B67] Sterling RK, Sulkowski MS (2004). Hepatitis C virus in the setting of HIV or hepatitis B virus coinfection. Semin Liver Dis.

[B68] Benhamou Y, Bochet M, Thibault V (1999). Long-term incidence of hepatitis B virus resistance to lamivudine in human immunodeficiency virus-infected patients. Hepatology.

